# Rab1A promotes cancer metastasis and radioresistance through activating GSK-3β/Wnt/β-catenin signaling in nasopharyngeal carcinoma

**DOI:** 10.18632/aging.103829

**Published:** 2020-10-17

**Authors:** Xian-Zi Yang, Xi-Min Chen, Li-Si Zeng, Jin Deng, Lei Ma, Chuan Jin, Ren Wang, Meng-He Wang, Yue-Feng Wen, Xiao-Liang Wu, Hui-Yun Wang, Shu-Zhong Cui

**Affiliations:** 1 Department of Medical Oncology, Affiliated Cancer Hospital and Institute of Guangzhou Medical University, Guangzhou 510095, China; 2 Institute of Oncology, Affiliated Cancer Hospital and Institute of Guangzhou Medical University, Guangzhou 510095, China; 3 Department of Radiation Oncology, Affiliated Cancer Hospital and Institute of Guangzhou Medical University, Guangzhou 510095, China; 4 Department of Clinical Laboratory, Maternity and Children's Healthcare Hospital of Foshan, Foshan 528000, China; 5 State Key Laboratory of Oncology in South China, Collaborative Innovation Center for Cancer Medicine, Guangdong Key Laboratory of Nasopharyngeal Carcinoma Diagnosis and Therapy, Sun Yat-Sen University Cancer Center, Guangzhou 510060, China; 6 Department of Oncology, Guizhou Provincial People’s Hospital, Guiyang 550200, China; 7 State Key Laboratory of Oncology in South China, Collaborative Innovation Center for Cancer Medicine, Sun Yat-Sen University Cancer Center, Guangzhou 510060, China; 8 Department of Abdominal Surgery, Affiliated Cancer Hospital and Institute of Guangzhou Medical University, Guangzhou 510095, China

**Keywords:** nasopharyngeal carcinoma, Rab1A, metastasis, radioresistance, GSK-3β/Wnt/β-catenin signaling

## Abstract

Many articles have reported that Rab1A was overexpressed in a variety of human cancers and involved in tumor progression and metastasis. However, the biological function and molecular mechanism of Rab1A in nasopharyngeal carcinoma (NPC) remained unknown until now. Here we found that Rab1A overexpression is a common event and was positively associated with distant metastasis and poor prognosis of NPC patients. Functionally, Rab1A depletion inhibited the migration and EMT phenotype of NPC cells, whereas Rab1A overexpression led to the opposite effect. Furthermore, we reveal an important role for Rab1A protein in the induction of radioresistance via regulating homologous recombination (HR) signaling pathway. Mechanistically, Rab1A activated Wnt/β-catenin signaling by inhibiting the activity of GSK-3β via phosphorylation at Ser9. Then Wnt/β-catenin signaling induced NPC cells radioresistance and metastasis through nuclear translocation of β-catenin and transcription upregulation of HR pathway-related and EMT-related genes expression. In general, this study shows that Rab1A may serve as a potential biomarker for predicting prognosis in NPC patients. Targeting Rab1A and Wnt/β-catenin signaling may hold promise to overcome NPC radioresistance.

## INTRODUCTION

Compared with other types of human cancers, nasopharyngeal carcinoma (NPC) is a rare form of cancer globally, but it remains endemic and has higher incidence rates in south China (Guangdong and Hong Kong) and southeastern Asia [1, 2]. The dramatic improvement of overall prognosis of NPC patients is mainly attributable to more accurate radiotherapy (RT) technology during the past three decades [3]. However, about 20% of patients develop local recurrent disease after radiotherapy, and 15% to 30% of patients will experience treatment failure because of distant metastases [2, 4]. Therefore, local recurrence and distant metastasis after RT remain a major barrier to restrict further improvement of prognosis of patients with NPC [5]. Although several lines of evidences demonstrated that elevated levels of EBV DNA and EBV-encoded latent membrane protein 1 (LMP1) were closely associated with tumor recurrence and metastasis [3, 6], the predictive value of EBV DAN and LMP1 on radioresistance remain controversial [4]. Therefore, there is an urgent need to better understand the mechanism underlying radiosensitivity and metastasis and identify more reliable prognostic biomarkers and therapeutic strategy in the management of NPC.

Radiotherapy causes cell death or cell apoptosis primarily by inducing DNA double-strand break (DSBs) [7], but a minority of tumor cells exhibit increased DNA damage response (DDR) in response to increased DNA damage, then maintain genomic integrity and escape IR (ionizing radiation)-induced lethality [8]. DDR is considered as the most critical cause of radiation resistance and local recurrence in cancer cells [9]. Recent studies have reported that epithelial-mesenchymal transition (EMT)-related regulators ZEB1 and Twist promoted cancer radioresistance through activation of DDR [10, 11]. These findings suggested that EMT may play a pivotal role in the regulation of radiosensitivity of human cancer by complex network of signaling pathways.

Rab1A, a highly conserved small GTPase, predominantly regulates dynamic membrane trafficking between endoplasmic reticulum (ER) and Golgi apparatus [12]. Recently, Rab1A has been validated to be a pivotal oncogene that promotes the progression and metastasis of colorectal cancer (CRC) and hepatocellular carcinoma (HCC) by activating mTORC1 signaling [13, 14]. On the other hand, some studies reported that reduced expression of Rab1A was observed in androgen-independent prostate cancer, and knockdown Rab1A enhanced the proliferation ability of prostate cancer cells [15]. A possible explanation for these reported contradictory functions of Rab1A protein may be due to its distinct roles in specific tumor types and/or other oncogenic events [12]. Previous researches have shown that Rab1A expression was significantly upregulated in human head and neck squamous cell carcinoma (HNSCC) [16] and murine fibroblast L929 cells [17] exposed to IR. Above results suggest that Rab1A is closely associated with radiosensitivity and tumor metastasis. However, the precise molecular mechanisms of Rab1A in the regulation of radioresistance and cancer metastases in NPC have not yet been unravelled. Therefore, in this present study, we investigated the expression and clinical predictive value of Rab1A in NPC samples and assessed the potential biological functions of Rab1A *in vitro*, then explored the molecular basis involved in tumor metastasis and radioresistance.

## RESULTS

### The expression of Rab1A is commonly up-regulated in advanced NPC tissues

**Figure 1:**
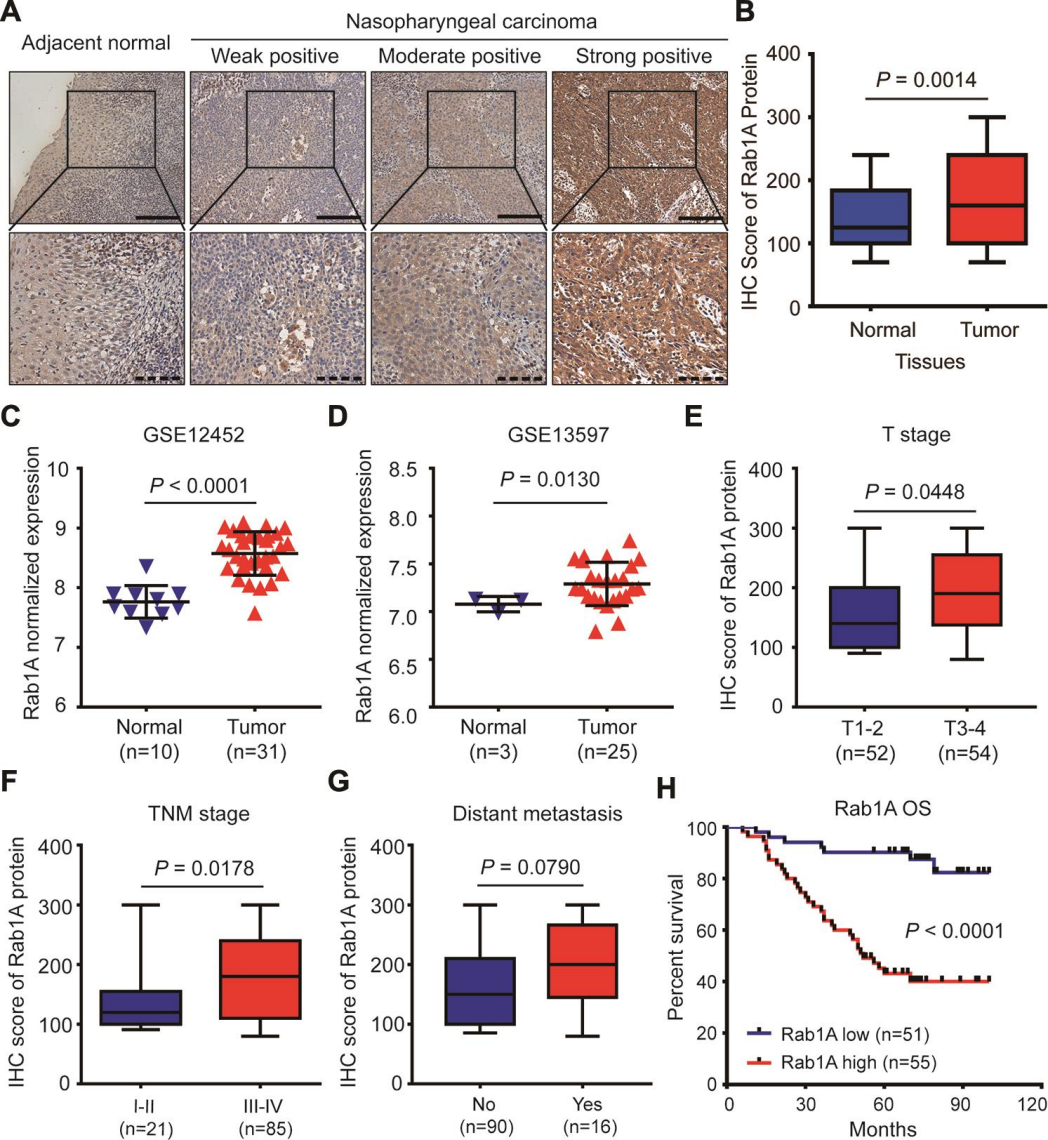
**Rab1A is highly expressed in NPC and positively correlates with poor prognosis of NPC patients.** (**A**) Shown are representative immunohistochemistry (IHC) staining of Rab1A in NPC and non-cancerous nasopharyngitis (NP) tissues. Lower panels represent magnified images of boxed area in the corresponding upper panel. The full line and dotted line scale represent 100 μm and 50 μm. (**B**) Comparsion of Rab1A expression between NPC tissues and NP tissues. The expression of Rab1A mRNA was upregulated in the Gene Expression Omnibus (GEO) database (**C**) GSE12452 and (**D**) GSE12597. (**E**–**G**) The expression of Rab1A in NPC patients with different T stage, different TNM stage or with/without distant metastasis. (**H**) Kaplan-Meier survival curve of OS in NPC patients with high (n=55) and low (n=51) levels of Rab1A expression. NPC patients with high expression of Rab1A have shorter OS than patients with low expression (*P* < 0.0001).

We firstly evaluated the expression of Rab1A in 106 NPC tissues and 54 non-cancerous nasopharyngitis (NP) tissues by immunohistochemistry (IHC). The representative images of NPC samples with different IHC scores are showed in Figure 1A. We found that Rab1A protein was mainly distributed in the cytoplasm of NPC cells (Figure 1A). Compared with non-cancerous tissues, the result showed that Rab1A expression was significantly higher in NPC tissues (*P* = 0.0014, Figure 1B). To validate our findings, we also downloaded two GEO datasets (GSE12452 and GSE 13597) of NPC mRNA expression profiles and analyzed Rab1A expression for further study [18, 19]. We confirmed that Rab1A was highly expressed in NPC samples in two above-mentioned datasets (Figure 1C and 1D). In different clinical subgroups, we found that Rab1A expression remarkably increased in patients with deep tumor invasion (T3-4) and advanced TNM stage (stage III-IV) (All *P* < 0.05, Figure 1E and 1F). And Rab1A marginally increased in patients with distant metastasis (*P* = 0.0790, Figure 1G). But no significantly statistical difference was observed between patients with and without lymphatic metastasis (*P* = 0.1019, Supplementary Figure 1A). Thus, these results indicated that Rab1A was commonly overexpressed in NPC tissues and may be involved in NPC progression and distant metastasis.

### Rab1A overexpression correlates with distant metastasis and poor prognosis of NPC patients

To investigate the clinical significance of Rab1A expression, these NPC patients were divided into high- and low-expression groups using the median IHC score (160) of Rab1A expression as the cutoff value. The association between Rab1A expression and clinicopathological characteristics was analyzed by Chi-square test. As shown in Table 1, high Rab1A expression was notably correlated with lymphatic metastasis (*P* = 0.043), distant metastasis (*P* = 0.045) and advanced clinical stages (*P* = 0.004). And high expression Rab1A level was marginally associated with deeper tumor invasion (*P* = 0.053). But there was no significantly statistical association between Rab1A expression and other clinicopathological parameters.

**Table 1:** Relationship between Rab1A expression and clinicopathological characteristics of NPC patients.

**Characteristics**	**Number of cases**	**Rab1A protein**		***P*-value ^a^**
		**Low (n=51)**	**High (n=55)**	
Gender				
Female	23	9 (39.1%)	14 (60.9%)	0.330
Male	83	42 (50.6%)	41 (49.4%)	
Age (years)				
< 45	60	29 (48.3%)	31 (51.7%)	0.959
≥ 45	46	22 (47.8%)	24 (52.2%)	
EA/IgA				
< 1:10	22	10 (45.5%)	12 (54.5%)	0.779
≥ 1:10	84	41 (48.8%)	43 (51.2%)	
VCA/IgA				
< 1:80	32	19 (59.4%)	13 (40.6%)	0.127
≥ 1:80	74	32 (43.2%)	42 (56.8%)	
EBV DNA copy				
< 4000	66	33 (50.0%)	33 (50.0%)	0.617
≥ 4000	40	18 (45.0%)	12 (55.0%)	
T stage				
T1-T2	52	30 (57.7%)	22 (42.3%)	0.053
T3-T4	54	21 (38.9%)	33 (61.1%)	
Lymphatic metastasis				
No	17	12 (70.6%)	5 (29.4%)	0.043
Yes	89	39 (43.8%)	50 (56.2%)	
Distant metastasis				
No	90	47 (52.2%)	43 (47.8%)	0.045
Yes	16	4 (25.0%)	12 (75.0%)	
TNM stage				
I-II	21	16 (76.2%)	5 (23.8%)	0.004
III-IV	85	35 (41.2%)	50 (58.8%)	

^a^ *P* < 0.05, Chi-square test. TNM stage, tumor-node-metastasis stage.

To reveal the prognostic value of Rab1A protein in this disease, Kaplan-Meier plots and the log-rank test were used to predict the outcomes of NPC patients with different Rab1A expression status. The result showed that patients with high Rab1A expression had significantly shorter 5-years overall survival (OS) rates (43.1% vs. 90.2%) than those with low Rab1A expression (*P* < 0.0001, Figure 1H). We further illustrated the relationship between various potential prognostic factors and clinical outcomes. Univariate Cox regression analysis indicated that distant metastasis, TNM stage and Rab1A expression were significant predictors of OS in NPC patients (All *P* < 0.05, Table 2). Multivariate analysis demonstrated that Rab1A was an independent risk predictor of OS for NPC patients (*P* < 0.001, Table 2). Additionally, distant metastasis was also an independent prognostic factor for OS (*P* = 0.007, Table 2). Taken together, our results suggested that Rab1A may serve as a novel prognostic biomarker for NPC patients.

**Table 2:** Univariate and multivariate analysis of various potential prognostic factors in NPC patients.

**Characteristics**	**Univariate analysis**		**Multivariate analysis**	
	***P* value ^a^**	**HR (95% CI)**	***P* value ^a^**	**HR (95% CI)**
Gender (Male vs. Female)	0.594	0.822 (0.401-1.688)		
Age. years (≥ 45 vs. < 45)	0.939	1.025 (0.544-1.931)		
EA/IgA (≥ 1:10 vs. < 1:10)	0.423	0.745 (0.363-1.530)		
VCA/IgA (≥ 1:80 vs. < 1:80)	0.829	1.080 (0.538-2.170)		
EBV DNA copy (≥ 4000 vs. < 4000)	0.152	0.600 (0.299-1.206)		
T stage (T3-T4 vs. T1-T2)	0.112	1.689 (0.885-3.221)		
Lymphatic metastasis (Yes vs. No)	0.206	1.951 (0.693-5.492)		
Distant metastasis (Yes vs. No)	< 0.001	3.552 (1.789-7.051)	0.007	2.642 (1.309-5.330)
TNM stage (III-IV vs. II-I)	0.014	5.911 (1.423-24.557)	0.121	3.159 (0.739-13.494)
Rab1A expression (high vs. low)	< 0.001	6.060 (2.658-13.819)	< 0.001	5.019 (2.173-11.592)

^a^ *P* < 0.05. TNM stage, tumor-node-metastasis stage; HR, hazard ratio; CI, confidence interval.

### Knockdown of Rab1A inhibits migration and enhances the radiosensitivity of NPC cells

**Figure 2:**
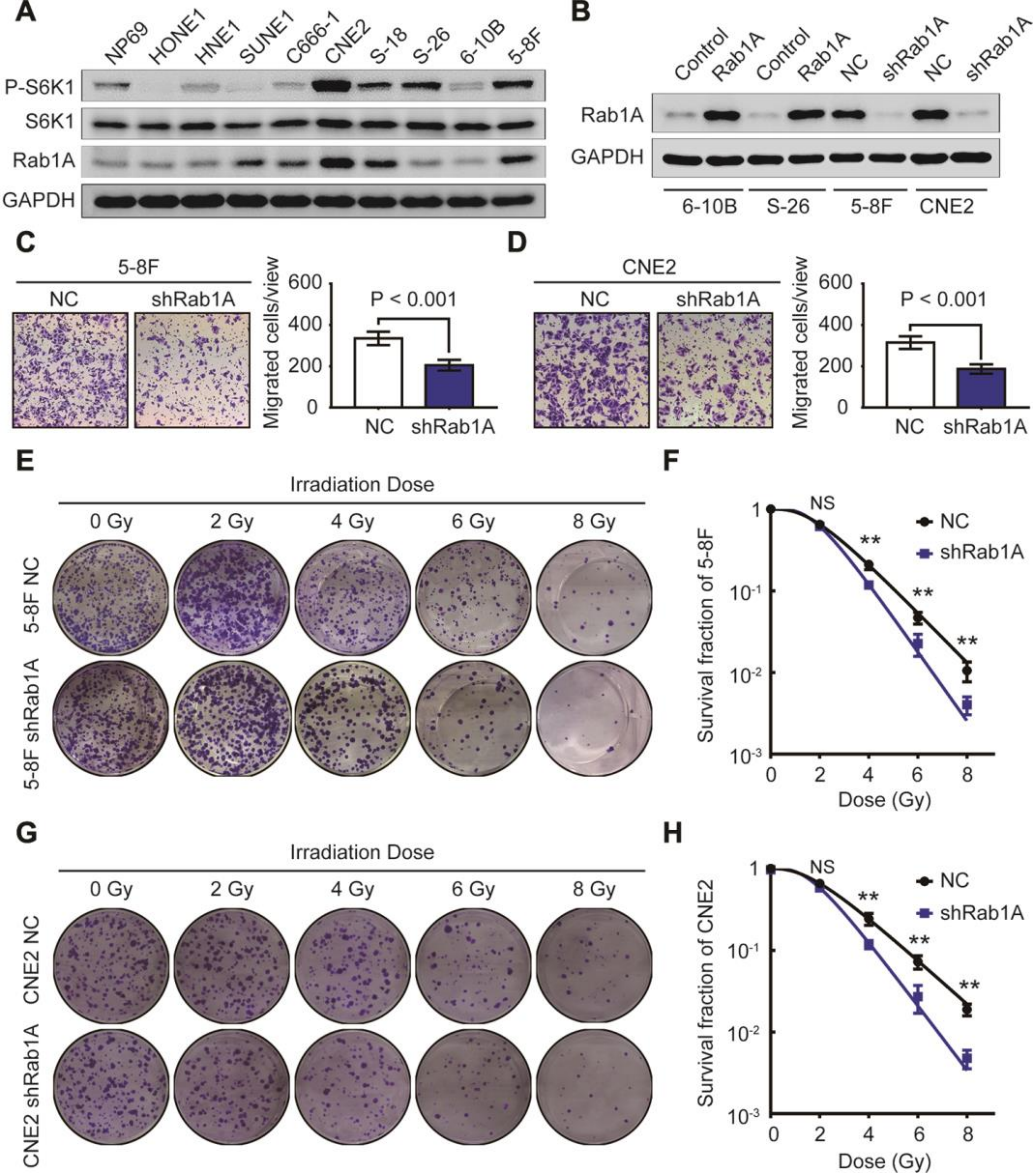
**Rab1A knockdown inhibits metastasis and enhances radiosensitivity in NPC cells *in vitro*.** (**A**) The expression of Rab1A, S6K1 and P-S6K1 proteins in 9 NPC cell lines and one human nasopharyngeal normal epithelium cell line NP69 is detected by western blotting. (**B**) Immunoblot analysis of Rab1A expression in 6-10B and S-26 cells transfected with Rab1A overexpression vector or 5-8F and CNE2 cells transfected with Rab1A shRNA. GAPDH served as a loading control. (**C** and **D**) The migration ability of 5-8F and CNE2 cells with Rab1A knockdown was evaluated by transwell assays. The left panels show representative images of cells that migrated through the PET membrane (magnification 200X). The right panel shows histograms of the data. Results are expressed as mean ± SD of three independent experiments. (**E**) Representative photos of 5-8F cells stably expressing Rab1A shRNA or a control shRNA cultured for 2 weeks after exposure to irradiation doses of 0, 2, 4, 6, 8 Gy, respectively. (**F**) Doses survival curve of 5-8F cells was constructed using the linear quadratic model. (**G**) Representative images of CNE2 cells transfected by Rab1A shRNA or scrambled shRNA (NC) cultured for 14 days after irradiation with the indicated Gy dose. (**H**) Doses survival curve of CNE2 cells was plotted using the linear quadratic model. Error bars: mean ± SD. ^**^P < 0.01, NS, no significant.

To investigate the biological consequences of dysregulated Rab1A in NPC cells, we first performed western blotting and RT-PCR in 9 NPC cell lines and one human nasopharyngeal normal epithelium cell line NP69. The results showed that compared with those in normal cell line, the expression of Rab1A protein and mRNA were significantly increased in 66.7% (6/9) and 88.9% (8/9) of NPC cell lines, respectively (Figure 2A and Supplementary Figure 1B). We also found that the expression of P-S6K1 protein was strikingly overexpressed in several NPC cell lines compared with in normal cell line NP69 (Figure 2A), but there was no significant correlation between Rab1A and P-S6K1 expression (*r* = 0.063, *P* = 0.863, Supplementary Figure 1C). We further used a previously validated lentiviral shRNA targeting Rab1A [13, 20] to transfect 5-8F and CNE2 cells with high endogenous Rab1A expression, and generated a stably overexpressing Rab1A in 6-10B and S-26 cells with low endogenous Rab1A level. The effective knockdown or overexpression of Rab1A in these NPC cell lines were testified by western blotting analysis (Figure 2B).

It has been well-known that the effects of radiotherapy are closely associated with NPC patients’ survival. As Rab1A overexpression was positively correlated with poor prognosis and distant metastasis, we reasoned that ectopic Rab1A expression affects the migratory capacity and radiosensitivity of NPC cells. In transwell assay, inhibition of Rab1A protein significantly decreased the number of migrated cells in 5-8F and CNE2 cell lines (both P < 0.001, Figure 2C and 2D). Next, 5-8F and CNE2 cells with or without Rab1A knockdown received irradiation doses of 0, 2, 4, 6, 8 Gy. At two weeks after exposure, colony formation assays showed that the ability to form survival foci in 5-8F and CNE2 cells was significantly attenuated by Rab1A inhibition. And this inhibition was obviously pronounced at 6 and 8 Gy (Figure 2E and 2G). The dose survival curves plotted by a linear quadratic model indicated that Rab1A knockdown increased the radiosensitivity of 5-8F and CNE2 cells (Figure 2F and 2H). Meanwhile, CCK-8 assay was conducted to measure the proliferative capacity of NPC cells with Rab1A knockdown before and after irradiation. The results indicated that down-regulation of Rab1A protein did not affect the proliferation of 5-8F and CNE2 cells treated without radiation (Supplementary Figure 1D and 1E). However, Rab1A knockdown significantly inhibited NPC cell proliferative abilities after 6 Gy of irradiation as a single dose (Supplementary Figure 1F and 1G). Taken together, our findings confirmed that reduced expression of Rab1A suppressed NPC metastasis and promoted the radiosensitivity of NPC cells.

### Rab1A overexpression enhances NPC metastasis and decreases their sensitivity to irradiation

**Figure 3:**
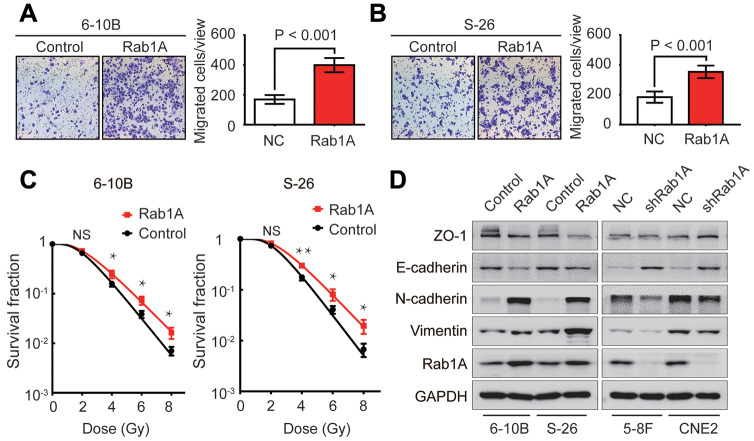
**Rab1A increases NPC cells metastasis, radioresistance and EMT phenotype.** (**A** and **B**) Migration assay of 6-10B and S-26 cells with Rab1A overexpression or a control vector by transwell assay. Left, representative images of invading cells. Right, quantification of cell invasion data. (**C**) Survival curves of 6-10B and S-26 cells with Rab1A overexpression or a control vector created by the linear quadratic equation. Means ± SD (n=3) are shown in A, B and C. NS, no significant, ^*^ *P* <0.05 and ^**^P < 0.01 versus control cells. (**D**) Western blotting assay to assess the expression of ZO-1, E-cadherin, N-cadherin and vimentin proteins in 6-10B and S-26 cells with Rab1A overexpression or 5-8F and CNE2 cells with Rab1A knockdown.

Following knockdown of Rab1A in NPC cells, we then artificially upregulated the expression levels of Rab1A protein in 6-10B and S-26 cells to observe its role in NPC metastasis and radioresistance. Transwell and clonogenic survival assays revealed that cell migration and radioresistance capacities were significantly reinforced after Rab1A overexpression (Figure 3A–3C). We further sought to explore whether Rab1A is implicated in the EMT process of NPC cells by western blotting assay. The results demonstrated that the EMT process was indeed triggered by aberrant expression of Rab1A in NPC cells. The gain of mesenchymal markers (N-cadherin and vimentin) and the loss of epithelial makers (E-cadherin and ZO-1) was observed in 6-10B and S-26 cells with Rab1A overexpression (Figure 3D). Conversely, Rab1A inhibition in 5-8F and CNE2 cells had the opposite effects on the EMT process (Figure 3D). Therefore, the results of these experiments demonstrated that enforced expression of Rab1A induced radioresistance and EMT in NPC cells.

### Rab1A expression reduces DSBs in NPC cells after irradiation

**Figure 4:**
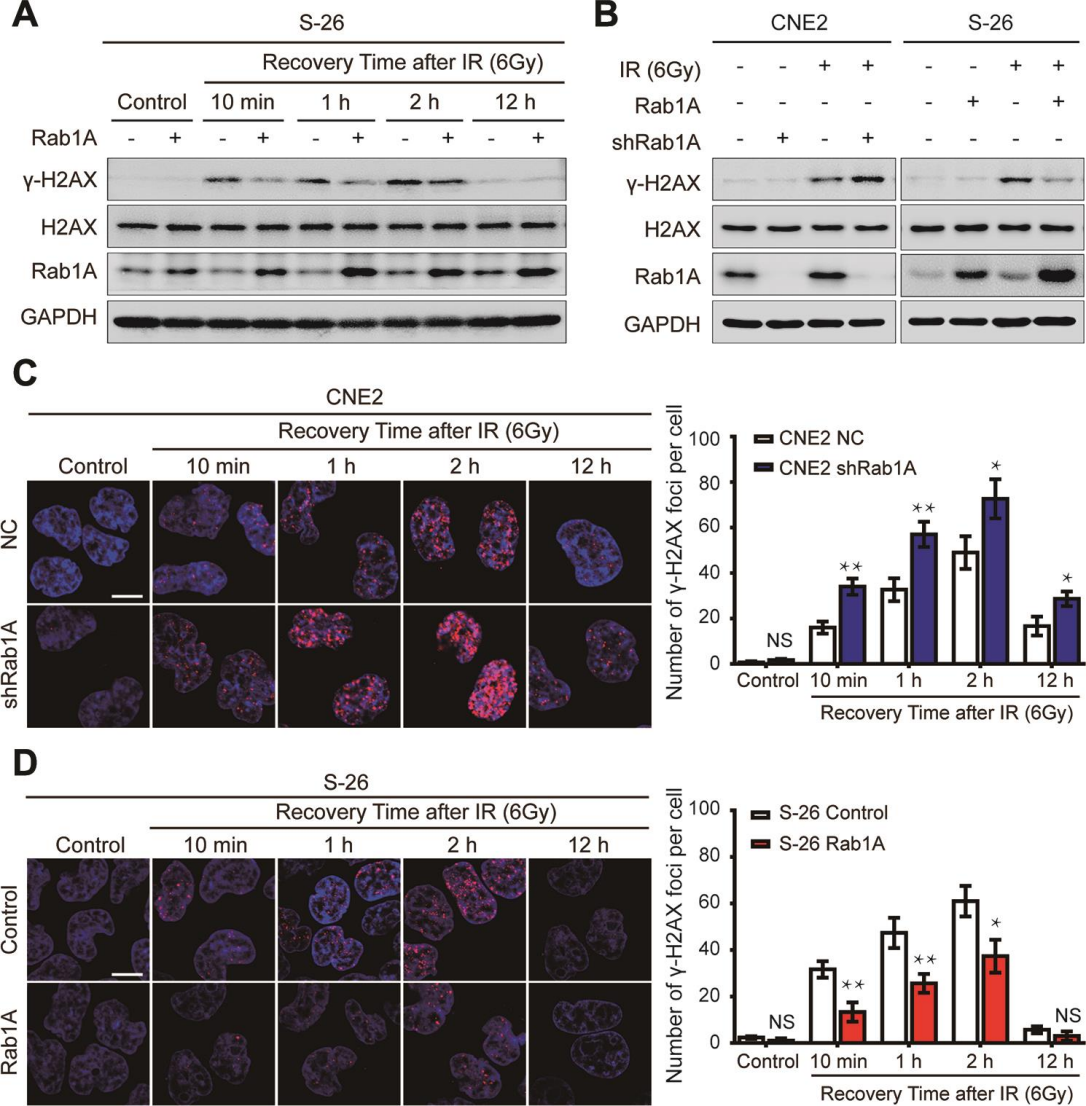
**Rab1A inhibits IR-inducible phosphorylation of serine 139 of H2AX (γ-H2AX) in NPC cells.** (**A**) Immunoblot analysis of 6 Gy IR-induced time-dependent changes of γ-H2AX, H2AX and Rab1A. Non-irradiated or irradiated S-26 cells with Rab1A overexpression or a control vector were collected at 10 min, 1 h, 2 h, 12 h after IR. (**B**) Immunoblot analysis of 6 Gy IR-induced time-dependent changes of γ-H2AX, H2AX and Rab1A in 6-10B and S-26 cells transfected with Rab1A overexpression vector or 5-8F and CNE2 cells transfected with Rab1A shRNA. GAPDH was used as a loading control. (**C** and **D**) Representative images of IF staining of γ-H2AX foci (red) in CNE2 cells with Rab1A knockdown or S-26 cells with Rab1A overexpression before IR exposure or after IR (6 Gy) for the indicated time. Nuclei were counterstained with DAPI (blue). Scale bars represent 5 μm. The number of foci for each time point was counted in 3 independent experiments (100 nuclei each). Histogram of the percentage of γ-H2AX foci was shown in right panel. For all quantitative results, the data are presented as the mean ± SD from three independent experiments. NS, no significant, ^*^P < 0.05 and ^**^P < 0.01.

Following the appearance of IR-induced DSBs, the phosphorylation of serine 139 of H2AX (γ-H2AX) rapidly increased and was recruited to the sites of DNA damage [21]. Thus, γ-H2AX is often regarded as a reliable marker of DNA damage, which is positively related with radiosensitivity [22]. To explore the effect of Rab1A expression on DSBs in NPC cells after irradiation, we firstly examined the expression of γ-H2AX protein in NPC cells by western blotting at different post-irradiation times. In S-26 cells, within the first 10 min of recovery following 6 Gy of irradiation, γ-H2AX expression rapidly increased and reached a peak at 2 hours. But no expression of γ-H2AX protein was detected before irradiation and γ-H2AX expression was almost eliminated after irradiated for 12 hours (Figure 4A). Interestingly, we also found that IR induced expression of Rab1A in a time-dependent manner (Figure 4A). Ectopic overexpression of Rab1A resulted in obvious downregulation of γ-H2AX expression at an early stage of post-irradiation recovery (Figure 4A). On the contrary, γ-H2AX expression was apparently higher in 6 Gy-irradiated CNE2 cells with Rab1A knockdown than that of control cells (Figure 4A). Before IR, γ-H2AX levels were very low and there were no differences between CNE2 cells expressing Rab1A shRNA or S-26 overexpressing Rab1A and those of control cells (Figure 4A and 4B).

We further performed immunofluorescent staining to quantify DSBs by analyzing the formation of γ-H2AX foci in CNE2 cells expressing Rab1A shRNA or S-26 overexpressing Rab1A, and comparing the results with those in control cells. It was shown that there was almost no γ-H2AX foci formation in all four groups before IR (Figure 4C and 4D). However, after treatment with 6 Gy radiation, the number of γ-H2AX foci significantly increased in all four groups (Figure 4C and 4D). Consistent with the results from western blotting assay, the percentage of γ-H2AX-positive cells was much higher in CNE2 cells with Rab1A knockdown, whereas Rab1A overexpression led to the opposite effect (Figure 4C and 4D). All these experiments demonstrated that Rab1A can decrease DNA damage accumulation induced by irradiation.

### Rab1A enhances NPC radioresistance by regulating the HR signaling pathway

**Figure 5:**
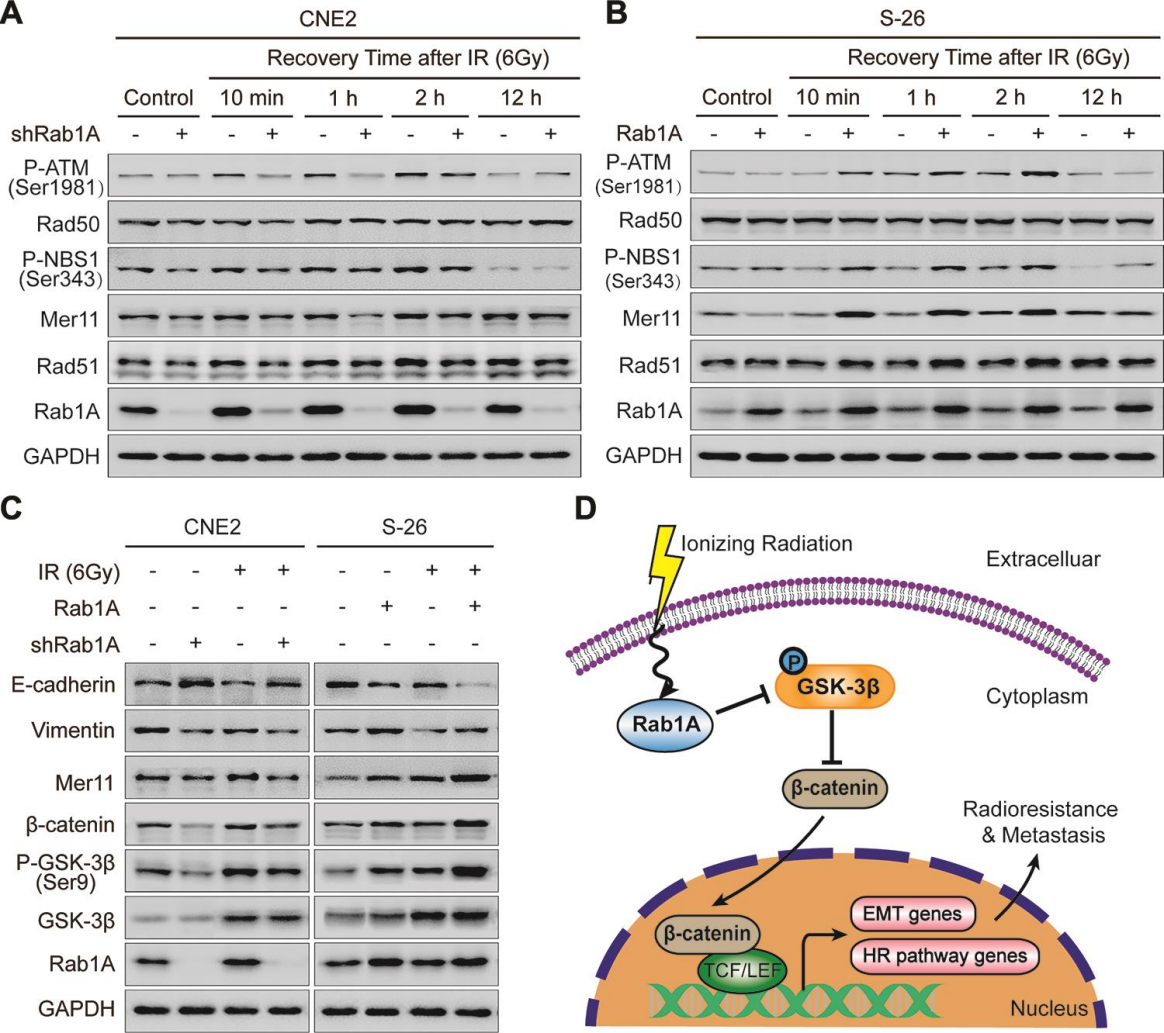
**Rab1A overexpression enhances NPC cells metastasis and HR pathway-mediated radioresistance through activating GSK-3β/Wnt/β-catenin signaling.** (**A** and **B**) Expression of typical HR pathway-related proteins, including Rad51, Mer11, P-NBS1(Ser343), Rad50 and P-ATM(Ser1981) based on western blotting, comparing the four groups of CNE2 cells transfected with Rab1A shRNA and S-26 cells transfected with Rab1A overexpression vector before IR exposure or after IR (6 Gy) for the indicated time. (**C**) Detection of Wnt pathway-related proteins (β-catenin, GSK-3β and P-GSK-3β), HR pathway protein (Mer11) and EMT-related proteins (E-cadherin and vimentin) in the four groups of CNE2 and S-26 cells treated with or without 6 Gy of IR. GAPDH was used as a loading control. Representative images are shown from three independent experiments. (**D**) Schematic diagram. Upon IR exposure, overexpression of Rab1A inhibits the activity of GSK-3β via phosphorylation at Ser9. The activation of Wnt/β-catenin signaling leads to nuclear translocation of β-catenin and transcription upregulation of HR pathway-related and EMT-related genes, in turn, induce NPC cells radioresistance and metastasis. The triangular arrows suggest positive regulation and the blunt arrows suggest negative regulation.

It has been well-established that IR-induced DSBs are predominantly repaired by two evolutionarily primary mechanisms: homologous recombination (HR) and non-homologous end joining (NHEJ) signaling pathways [23]. To explore the underlying mechanism by which Rab1A reduces DNA damage, we detected the expression levels of HR and NHEJ pathway-related proteins at different times after irradiation with 6 Gy. After irradiation, knockdown of Rab1A in CNE2 cells significantly inhibited the expression of Rad51, Mer11, P-NBS1(Ser343) and P-ATM (Ser1981) proteins compared with those of control cells. And the most obvious inhibitory effect was investigated at 1 hour after IR (Figure 5A). But the expression of Rad50 protein did not significantly differ between CNE2 cells expressing Rab1A shRNA and a control shRNA (Figure 5A). Furthermore, we found that S-26 cells with stable Rab1A overexpression displayed the opposite results (Figure 5B). Besides, the impact of anomalous expression of Rab1A on the levels of NHEJ pathway-related proteins (DNA-PKcs and Ku80) was evaluated by western blotting assay after irradiation. Unfortunately, there were no significantly differences in DNA-PKcs and Ku80 proteins expression between CNE2 cells expressing Rab1A shRNA or S-26 overexpressing Rab1A and those of control cells (Supplementary Figure 2A and 2B). Collectively, our results suggested that the HR pathway could be involved in Rab1A-mediated radioresistance in NPC cells.

### Rab1A promotes NPC metastasis and radioresistance via mainly stimulating GSK-3β/Wnt/β-catenin signaling

It is generally accepted that the canonical Wnt signaling pathway is tightly linked with tumor metastasis, EMT phenotype and radioresistance of NPC cells [24, 25]. So we further examined the expression levels of Wnt-related and EMT-related proteins in NPC cells with different Rab1A expression levels before and after irradiation. As expected, depletion of Rab1A in CNE2 cells decreased the abundance of β-catenin and Ser9 glycogen synthase kinase 3β (GSK-3β) phosphorylation, an event known to inhibit GSK-3β kinase activity. This inhibitory effect was more substantial after irradiation with 6 Gy (Figure 5C). The expression of total GSK-3β protein also increased after IR, whereas no significant change was observed between CNE2 cells expressing Rab1A shRNA and control cells. Simultaneously, CEN2 cells with Rab1A inhibition displayed lower expression of vimentin and Mer11 proteins and higher expression of E-cadherin protein compared with those of control cells before and after IR (Figure 5C). In contrast, elevated expression of Rab1A resulted in the promotive effects in S-26 cells treated with or without irradiation (Figure 5C). Primed by our findings, we suggested that IR could increases Rab1A expression, then stimulates Wnt/β-catenin signaling by inhibition of GSK-3β activity via phosphorylation at Ser9. The activation of Wnt/β-catenin signaling leads to nuclear translocation of β-catenin and transcription upregulation of HR pathway-related and EMT-related genes, in turn, induce NPC cells radioresistance and metastasis (Figure 5D).

## DISCUSSION

In the current study, we confirmed the overexpression of Rab1A in NPC samples and its positive correlation with tumor metastasis of NPC patients. These results were consistent with previous findings [13, 14, 26–28], suggesting that Rab1A may play a commonly oncogenic role in certain types of cancers. In terms of its cellular effects, some studies showed that Rab1A promoted cellular proliferation, invasion and EMT process through activation of mTOR signaling in several cancers [13, 14, 27]. Consistently, Rab1A overexpression enhanced the cell migratory capacity and EMT phenotype of NPC cells.

As we all known, the most cytotoxic lesion of IR-induced DNA damage is DSBs which is closely correlated with cancer radiosensitivity [29]. In the initial step of DDR, the phosphorylation of H2AX (γ-H2AX) exerts a critical role in recognizing the DSB sites, which is a recognized biomarker of DSBs [30]. Our results provided the first evidence that Rab1A significantly decreased γ-H2AX expression and potentiated DSBs repair in NPC cells exposed to IR. DSBs are repaired by two primary pathways: homologous recombination (HR) and non-homologous end joining (NHEJ) [31]. A recent study reported that the activation of HR signaling pathway was positively correlated with NPC radioresistance [32]. As expected, our results demonstrated that HR signaling pathway (not NHEJ pathway) is responsible for the regulation of Rab1A-mediated radioresistance in NPC cells, which was consistent with previous results [32].

The canonical Wnt/β-catenin signaling is reported to be implicated in cancer stemness, EMT and radioresistance in several human cancers [33]. The core proteins of this signaling is β-catenin which not only links E-cadherin and α-catenin to regulate cell-to-cell adhesion and cell migration, but also acts as a key downstream cofactor of this pathway that results in the transcriptional activation of the β-catenin target genes and cell survival [34]. What’s more, the known function of cytoplasmic GSK-3β is the regulation of β-catenin degradation by phosphorylation [35]. GSK-3β also produced radioresistance of pancreatic cancer cells by a β-catenin dependent mechanism [36]. So it seems plausible that both β-catenin target genes and GSK-3β participate in Wnt-induced metastasis and radioresistance. As anticipated, our data showed that Rab1A increased total β-catenin expression via promoting Ser9 phosphorylation and suppressing GSK-3β activity, then enhanced the expression of EMT-related and HR pathway-related proteins under irradiation condition.

Interestingly, a previous study demonstrated that EMT contributed to acquired chemoresistance in breast cancer cells [37]. Our findings showed that Rab1A could simultaneously increase EMT and radioresistance through activating Wnt signaling. But whether EMT helps NPC cells to resist IR damage is still largely unknown. Besides, considering previous studies in CRC and HCC [13, 14], whether Rab1A may bind certain proteins or mediate other pathways (including mTORC1 signaling) to regulate NPC cells metastasis and radioresistance remains to be determined. We plan to further answer these questions in our future studies.

In summary, our data indicated that Rab1A overexpression is a common event and an independent risk predictor for NPC patients. Rab1A exerts its promotive effect on NPC migration and EMT process. We also find an important role for Rab1A in the induction of radioresistance by HR signaling pathway. Our study defines a mechanism for the function of Rab1A that activates GSK-3β/β-catenin/Wnt signaling pathway, then promotes NPC metastasis and radioresistance. Therefore, Rab1A may serve as a promising biomarker for predicting outcome and a potential therapeutic target for overcoming NPC radioresistance.

## MATERIALS AND METHODS

### Patients and samples

This study was reviewed and approved by the institutional review board and ethics committee of Affiliated Cancer Hospital and Institute of Guangzhou Medical University. Written informed consent was obtained from all patients or their guardians. The formalin-fixed, paraffin-embedded tumor and non-cancerous nasopharyngitis (NP) tissues samples were collected from 106 primary NPC patients with stage I-IV between January 2011 and December 2014. None of patients enrolled in this study were given local or systemic treatment before biopsy sampling. All of NPC samples had been confirmed pathologically by two experienced pathologists. The clinical and pathological information of these NPC patients were listed in Table 1.

### Bioinformatics analysis

To investigate the expression of Rab1A in NPC, we downloaded two sets of mRNA expression profiles for NPC, GSE12452 and GSE 13597 from GEO database [18, 19]. The expression of Rab1A mRNA was analyzed by the Significant Analysis of Microarray (SAM) software.

### Cell lines and cell transfection

Nine human NPC cell lines including HONE1, HNE1, SUNE1, C666-1, CNE2, S-18, S-26, 6-10B and 5-8F as well as one human nasopharyngeal normal epithelium cell line NP69 were maintained in our laboratory [38]. NPC cell lines were cultured in RPMI-1640 medium supplemented with 10% fetal bovine serum (Hyclone, Logan, UT, USA). The NP69 cell line was grown in defined-KSFM medium supplemented with EGF (Invitrogen, Carlsbad, CA, USA). All cell lines were cultured in a humidified cell incubator at 37°C with an atmosphere of 5% CO_2_.

The lentivirus-containing short hairpin RNA (shRNA) targeting Rab1A were purchased from Thermo Fisher Scientific and the sequence of Rab1A shRNA was described previously [14]. Human Rab1A mRNA sequence (GenBank, NM_004161.4) were synthesized and cloned into pGLV5/GFP lentiviral plasmids. Rab1A expression lentiviral vector was supplied by GenePharma, Co, Ltd (Shanghai, China). NPC cells were transfected with the above-mentioned lentiviral vectors. Stable NPC cell lines expressing Rab1A shRNA or overexpression Rab1A were selected using 4 μg/ml puromycin for two weeks according to the manufacturer’s instructions.

### Immunohistochemistry (IHC) staining

All formalin-fixed, paraffin-embedded samples were cut into 4 μm thick sections. IHC staining was conducted using standard streptavidin-peroxidase complex method as described previously [39]. Immunohistochemical score was independently assessed by two pathologists without knowledge of patient characteristics. For IHC score, the percentage (0-100 %) of stained tumor cells was multiplied by the intensity (0, 1, 2, or 3) to achieve a total score between 0 and 300.

### Real-time PCR (RT-PCR)

Total RNA from NPC cells was isolated using TRIzol reagent (Invitrogen) as per the manufacturer’s recommendation. For first-strand cDNA synthesis, 1 μg of Total RNA was reversed transcribed using the Moloney Leukemia Virus Reverse Transcriptase Kit (Promega, Madison, WI, USA). Real-time PCR was carried out by using the SYBR Green Mix Kit (Promega) in a Roche Lightcycler 96 real time RCR machine (Roche Diagnostics, Indianapolis, IN, USA). The relative expression of target genes was calculated using the comparative 2^-ΔΔCT^ method. The sequences of RT-PCR primers are as follows: Rab1A forward primer: 5’- CAGCAGGCCAGGAAAGATT -3’, reverse primer: 5’- GGTCAGATCACATTTGTTCCCTA-3’; GAPDH forward primer: 5’-CTCCTCCTGTTCGACAGTCAGC-3’, reverse primer: 5’-CCCAATACGACCAAATCCGTT-3’. All samples were amplified in triplicate and GAPDH was detected as an internal control.

### CCK8 assay and transwell assay

For the CCK8 assay, cells transfected with Rab1A shRNA were seeded into 96-well plates at a density of 1000 cells/well for 24 h incubation. Then cells treated with or without 6 Gy of irradiation were detected by CCK8 Cell Counting Kit (Dojindo, Tokyo, Japan) at a regular time every day. After a further 2 h incubation, the OD value was measured at 450 nm by a microplate reader (Bio-Rad, Hercules, CA). For the transwell assay, 4 × 10^4^ cells were suspended by serum-free medium and seeded in the upper chamber (BD Biosciences). RPMI-1640 containing 20% FBS was added to the lower chamber of each well in a 24-well plate. After 24h incubation, those non-invading cells of upper chamber were removed by a cotton swab. Cells on the lower surface of the scrubbed membranes were fixed with methanol and stained with 0.1 % crystal violet. Five random fields of each chamber were counted under a microscope. Experiments were performed in triplicate.

### Clonogenic cell survival assays

Cell transfected with Rab1A shRNA or Rab1A overexpression vector or those of control plasmids were seeded in 6-well plates (200, 500, 1000, 5000 and 10000 cells per well in triplicate) and incubated for 24h for seeding. They were then treated in a single session with radiation doses of 0, 2, 4, 6, 8 Gy. Cells were cultured for 2 weeks at 37°C with 5% CO_2_ to allow the colonies to form, then stained with 0.1 % crystal violet. The colonies containing more than 50 cells were identified as surviving colonies. Finally, the data were fitted into the linear quadratic model to plot the clonogenic cell survival curves to estimate the radiosensitivity of these cells.

### Immunofluorescence (IF) assay

Immunofluorescence (IF) assay was performed as described previously [40]. Briefly, cell were seeded on glass coverslips in 24-well plates and exposed to a radiation dose of 6 Gy. They were then incubated for specified times (0, 10 min, 1 h, 2 h and 12 h) after IR. Cells were fixed with 4% paraformaldehyde (PFA) for 20 min, permeabilized with 0.25% Triton X-100 for 10 min and blocked in 4% bovine serum albumin for 1 h. Cells were incubated with primary antibody (4°C, overnight) against γ-H2AX (Cell Signaling Technology, MA, USA) followed by incubation with the fluorescent secondary antibody. Then cells were stained with DAPI (Life Technologies, Carlsbad, CA, USA) to visualize nuclear DNA. Finally, images of coverslips were captured under a confocal laser scanning microscope (FV1000; Olympus, Tokyo, Japan).

### Western blotting

Whole-cell lysates were harvested using RIPA buffer (Cell Signaling Technology) containing protease inhibitor cocktail, the PhosSTOP phosphatase inhibitors and PMSF (Roche, Basel, Switzerland). 30 μg of lysate protein were then fractionated in SDS-polyacrylamide gels and transferred onto polyvinylidene fluoride membranes (PVDF, Millipore, USA). After blocking antigen, the membranes were incubated with primary antibodies and the corresponding secondary antibodies. The primary antibody used in this assay included Rab1A (Santa Cruz, USA) and GAPDH, S6K1, P-S6K1(T398), E-cadherin, N-cadherin, Vimentin, ZO-1, H2AX, γ-H2AX, Rad50, Mer11, P-NBS1(Ser343), P-ATM(Ser1981), Ku80, DNA-PKcs, β-catenin, GSK-3β, P-GSK-3β(Ser9) (Cell Signaling Technology) and Rad51 (Proteintech Group, USA). Finally, proteins were visualized with Western Lightning Chemiluminescence Reagent Plus (PerkinElmer, Waltham, MA, USA).

### Statistical analysis

Statistical analyses were conducted using SPSS 23.0 (SPSS, Chicago, IL, USA) or GraphPad PrismV7 (GraphPad, La Jolla, CA, USA). The Chi-square test was used for correlation analysis between clinicopathological characteristics of patients and Rab1A expression. Survival curves were plotted by the Kaplan-Meier method and compared using the log-rank test. Student’s *t* tests or χ^2^ tests were performed to detect significant differences between tow groups of data. The dose survival curve was calculated by a linear quadratic model (*Y* = exp(-(a*x + b*(*x*^2)))). All the quantitative data presented were the mean ± SD from at least three independent samples. *P* values less than 0.05 were considered statistically significant.

## Supplementary Material

Supplementary Figures

## References

[1] Torre LA, Bray F, Siegel RL, Ferlay J, Lortet-Tieulent J, Jemal A. Global cancer statistics, 2012. CA Cancer J Clin. 2015; 65:87–108. 10.3322/caac.21262 25651787

[2] Lee AW, Ma BB, Ng WT, Chan AT. Management of nasopharyngeal carcinoma: current practice and future perspective. J Clin Oncol. 2015; 33:3356–64. 10.1200/JCO.2015.60.9347 26351355

[3] Chua ML, Wee JT, Hui EP, Chan AT. Nasopharyngeal carcinoma. Lancet. 2016; 387:1012–24. 10.1016/S0140-6736(15)00055-0 26321262

[4] Liu SC, Tsang NM, Chiang WC, Chang KP, Hsueh C, Liang Y, Juang JL, Chow KP, Chang YS. Leukemia inhibitory factor promotes nasopharyngeal carcinoma progression and radioresistance. J Clin Invest. 2013; 123:5269–83. 10.1172/JCI63428 24270418 PMC3859424

[5] Zhang L, Huang Y, Hong S, Yang Y, Yu G, Jia J, Peng P, Wu X, Lin Q, Xi X, Peng J, Xu M, Chen D, et al Gemcitabine plus cisplatin versus fluorouracil plus cisplatin in recurrent or metastatic nasopharyngeal carcinoma: a multicentre, randomised, open-label, phase 3 trial. The Lancet. 2016; 388:1883–1892. 10.1016/S0140-6736(16)31388-5 27567279

[6] Li G, Wu Z, Peng Y, Liu X, Lu J, Wang L, Pan Q, He ML, Li XP. MicroRNA-10b induced by epstein-barr virus-encoded latent membrane protein-1 promotes the metastasis of human nasopharyngeal carcinoma cells. Cancer Lett. 2010; 299:29–36. 10.1016/j.canlet.2010.07.021 20732742

[7] Frankenberg-Schwager M, Frankenberg D, Blöcher D, Adamczyk C. Effect of dose rate on the induction of DNA double-strand breaks in eucaryotic cells. Radiat Res. 1981; 87:710–17. 10.2307/3575532 7025087

[8] Jameel JK, Rao VS, Cawkwell L, Drew PJ. Radioresistance in carcinoma of the breast. Breast. 2004; 13:452–60. 10.1016/j.breast.2004.08.004 15563851

[9] Morgan MA, Lawrence TS. Molecular pathways: overcoming radiation resistance by targeting DNA damage response pathways. Clin Cancer Res. 2015; 21:2898–904. 10.1158/1078-0432.CCR-13-3229 26133775 PMC4494107

[10] Zhang P, Wei Y, Wang L, Debeb BG, Yuan Y, Zhang J, Yuan J, Wang M, Chen D, Sun Y, Woodward WA, Liu Y, Dean DC, et al. ATM-mediated stabilization of ZEB1 promotes DNA damage response and radioresistance through CHK1. Nat Cell Biol. 2014; 16:864–75. 10.1038/ncb3013 25086746 PMC4150825

[11] Zhang L, Su B, Sun W, Li W, Luo M, Liu D, Mei Q, Long G, Hu G, Hu G. Twist1 promotes radioresistance in nasopharyngeal carcinoma. Oncotarget. 2016; 7:81332–40. 10.18632/oncotarget.12875 27793033 PMC5348396

[12] Yang XZ, Li XX, Zhang YJ, Rodriguez-Rodriguez L, Xiang MQ, Wang HY, Zheng XF. Rab1 in cell signaling, cancer and other diseases. Oncogene. 2016; 35:5699–704. 10.1038/onc.2016.81 27041585 PMC5396462

[13] Xu BH, Li XX, Yang Y, Zhang MY, Rao HL, Wang HY, Zheng XF. Aberrant amino acid signaling promotes growth and metastasis of hepatocellular carcinomas through Rab1A-dependent activation of mTORC1 by Rab1A. Oncotarget. 2015; 6:20813–28. 10.18632/oncotarget.5175 26308575 PMC4673231

[14] Thomas JD, Zhang YJ, Wei YH, Cho JH, Morris LE, Wang HY, Zheng XF. Rab1A is an mTORC1 activator and a colorectal oncogene. Cancer Cell. 2016; 30:181–82. 10.1016/j.ccell.2016.06.014 27479033

[15] Sun T, Wang X, He HH, Sweeney CJ, Liu SX, Brown M, Balk S, Lee GS, Kantoff PW. MiR-221 promotes the development of androgen independence in prostate cancer cells via downregulation of HECTD2 and RAB1A. Oncogene. 2014; 33:2790–800. 10.1038/onc.2013.230 23770851 PMC3883998

[16] Jelonek K, Wojakowska A, Marczak L, Muer A, Tinhofer-Keilholz I, Lysek-Gladysinska M, Widlak P, Pietrowska M. Ionizing radiation affects protein composition of exosomes secreted in vitro from head and neck squamous cell carcinoma. Acta Biochim Pol. 2015; 62:265–72. 10.18388/abp.2015_970 26098714

[17] Szkanderová S, Port M, Stulík J, Hernychová L, Kasalová I, Van Beuningen D, Abend M. Comparison of the abundance of 10 radiation-induced proteins with their differential gene expression in L929 cells. Int J Radiat Biol. 2003; 79:623–33. 10.1080/09553000310001606821 14555345

[18] Sengupta S, den Boon JA, Chen IH, Newton MA, Dahl DB, Chen M, Cheng YJ, Westra WH, Chen CJ, Hildesheim A, Sugden B, Ahlquist P. Genome-wide expression profiling reveals EBV-associated inhibition of MHC class I expression in nasopharyngeal carcinoma. Cancer Res. 2006; 66:7999–8006. 10.1158/0008-5472.CAN-05-4399 16912175

[19] Bose S, Yap LF, Fung M, Starzcynski J, Saleh A, Morgan S, Dawson C, Chukwuma MB, Maina E, Buettner M, Wei W, Arrand J, Lim PV, et al. The ATM tumour suppressor gene is down-regulated in EBV-associated nasopharyngeal carcinoma. J Pathol. 2009; 217:345–52. 10.1002/path.2487 19142888

[20] Thomas JD, Zhang YJ, Wei YH, Cho JH, Morris LE, Wang HY, Zheng XF. Rab1A is an mTORC1 activator and a colorectal oncogene. Cancer Cell. 2014; 26:754–69. 10.1016/j.ccell.2014.09.008 25446900 PMC4288827

[21] Matthaios D, Hountis P, Karakitsos P, Bouros D, Kakolyris S. H2AX a promising biomarker for lung cancer: a review. Cancer Invest. 2013; 31:582–99. 10.3109/07357907.2013.849721 24164298

[22] Lu J, Tang M, Li H, Xu Z, Weng X, Li J, Yu X, Zhao L, Liu H, Hu Y, Tan Z, Yang L, Zhong M, et al. EBV-LMP1 suppresses the DNA damage response through DNA-PK/AMPK signaling to promote radioresistance in nasopharyngeal carcinoma. Cancer Lett. 2016; 380:191–200. 10.1016/j.canlet.2016.05.032 27255972

[23] Curtin NJ. DNA repair dysregulation from cancer driver to therapeutic target. Nat Rev Cancer. 2012; 12:801–17. 10.1038/nrc3399 23175119

[24] Li G, Liu Y, Su Z, Ren S, Zhu G, Tian Y, Qiu Y. MicroRNA-324-3p regulates nasopharyngeal carcinoma radioresistance by directly targeting Wnt2B. Eur J Cancer. 2013; 49:2596–607. 10.1016/j.ejca.2013.03.001 23583221

[25] Zhang J, Wen X, Ren XY, Li YQ, Tang XR, Wang YQ, He QM, Yang XJ, Sun Y, Liu N, Ma J. YPEL3 suppresses epithelial-mesenchymal transition and metastasis of nasopharyngeal carcinoma cells through the Wnt/β-catenin signaling pathway. J Exp Clin Cancer Res. 2016; 35:109. 10.1186/s13046-016-0384-1 27400785 PMC4940860

[26] Wang X, Liu F, Qin X, Huang T, Huang B, Zhang Y, Jiang B. Expression of Rab1A is upregulated in human lung cancer and associated with tumor size and T stage. Aging (Albany NY). 2016; 8:2790–98. 10.18632/aging.101087 27902464 PMC5191870

[27] Xu H, Qian M, Zhao B, Wu C, Maskey N, Song H, Li D, Song J, Hua K, Fang L. Inhibition of RAB1A suppresses epithelial-mesenchymal transition and proliferation of triple-negative breast cancer cells. Oncol Rep. 2017; 37:1619–26. 10.3892/or.2017.5404 28184936

[28] Shao X, Cheng Z, Xu M, Mao J, Wang J, Zhou C. Prognosis, significance and positive correlation of Rab1A and p-S6K/Gli1 expression in gastric cancer. Anticancer Agents Med Chem. 2019; 19:1359–67. 10.2174/1871520619666190416110851 31038077

[29] Wei F, Tang L, He Y, Wu Y, Shi L, Xiong F, Gong Z, Guo C, Li X, Liao Q, Zhang W, Ni Q, Luo J, et al. BPIFB1 (LPLUNC1) inhibits radioresistance in nasopharyngeal carcinoma by inhibiting VTN expression. Cell Death Dis. 2018; 9:432. 10.1038/s41419-018-0409-0 29568064 PMC5864881

[30] Olive PL. Retention of γH2AX foci as an indication of lethal DNA damage. Radiother Oncol. 2011; 101:18–23. 10.1016/j.radonc.2011.05.055 21704409

[31] Chowdhury D, Choi YE, Brault ME. Charity begins at home: non-coding RNA functions in DNA repair. Nat Rev Mol Cell Biol. 2013; 14:181–89. 10.1038/nrm3523 23385724 PMC3904369

[32] Wang Z, Zuo W, Zeng Q, Li Y, Lu T, Bu Y, Hu G. The homologous recombination repair pathway is associated with resistance to radiotherapy in nasopharyngeal carcinoma. Int J Biol Sci. 2020; 16:408–19. 10.7150/ijbs.37302 32015678 PMC6990897

[33] Zhao Y, Tao L, Yi J, Song H, Chen L. The role of canonical Wnt signaling in regulating radioresistance. Cell Physiol Biochem. 2018; 48:419–32. 10.1159/000491774 30021193

[34] Clevers H, Nusse R. Wnt/β-catenin signaling and disease. Cell. 2012; 149:1192–205. 10.1016/j.cell.2012.05.012 22682243

[35] Zeng X, Tamai K, Doble B, Li S, Huang H, Habas R, Okamura H, Woodgett J, He X. A dual-kinase mechanism for Wnt co-receptor phosphorylation and activation. Nature. 2005; 438:873–77. 10.1038/nature04185 16341017 PMC2100418

[36] Watson RL, Spalding AC, Zielske SP, Morgan M, Kim AC, Bommer GT, Eldar-Finkelman H, Giordano T, Fearon ER, Hammer GD, Lawrence TS, Ben-Josef E. GSK3beta and beta-catenin modulate radiation cytotoxicity in pancreatic cancer. Neoplasia. 2010; 12:357–65. 10.1593/neo.92112 20454507 PMC2864473

[37] Fischer KR, Durrans A, Lee S, Sheng J, Li F, Wong ST, Choi H, El Rayes T, Ryu S, Troeger J, Schwabe RF, Vahdat LT, Altorki NK, et al. Epithelial-to-mesenchymal transition is not required for lung metastasis but contributes to chemoresistance. Nature. 2015; 527:472–76. 10.1038/nature15748 26560033 PMC4662610

[38] Wang MH, Zhou XM, Zhang MY, Shi L, Xiao RW, Zeng LS, Yang XZ, Zheng XF, Wang HY, Mai SJ. BMP2 promotes proliferation and invasion of nasopharyngeal carcinoma cells via mTORC1 pathway. Aging (Albany NY). 2017; 9:1326–40. 10.18632/aging.101230 28455969 PMC5425130

[39] Yang XZ, Cui SZ, Zeng LS, Cheng TT, Li XX, Chi J, Wang R, Zheng XF, Wang HY. Overexpression of Rab1B and MMP9 predicts poor survival and good response to chemotherapy in patients with colorectal cancer. Aging (Albany NY). 2017; 9:914–31. 10.18632/aging.101200 28316326 PMC5391239

[40] Yang XZ, Cheng TT, He QJ, Lei ZY, Chi J, Tang Z, Liao QX, Zhang H, Zeng LS, Cui SZ. LINC01133 as ceRNA inhibits gastric cancer progression by sponging miR-106a-3p to regulate APC expression and the Wnt/β-catenin pathway. Mol Cancer. 2018; 17:126. 10.1186/s12943-018-0874-1 30134915 PMC6106894

