# Supplementary material for: Rab1A promotes cancer metastasis and radioresistance through activating GSK-3β/Wnt/β-catenin signaling in nasopharyngeal carcinoma
Source: Aging (Albany NY). 2020 Oct 17;12(20):20380–95. doi: 10.18632/aging.103829 (PMC7655155; doi:10.18632/aging.103829)
Supplement: Supplementary Figures [file aging-12-103829-s001..pdf]

SUPPLEMENTARY FIGURES

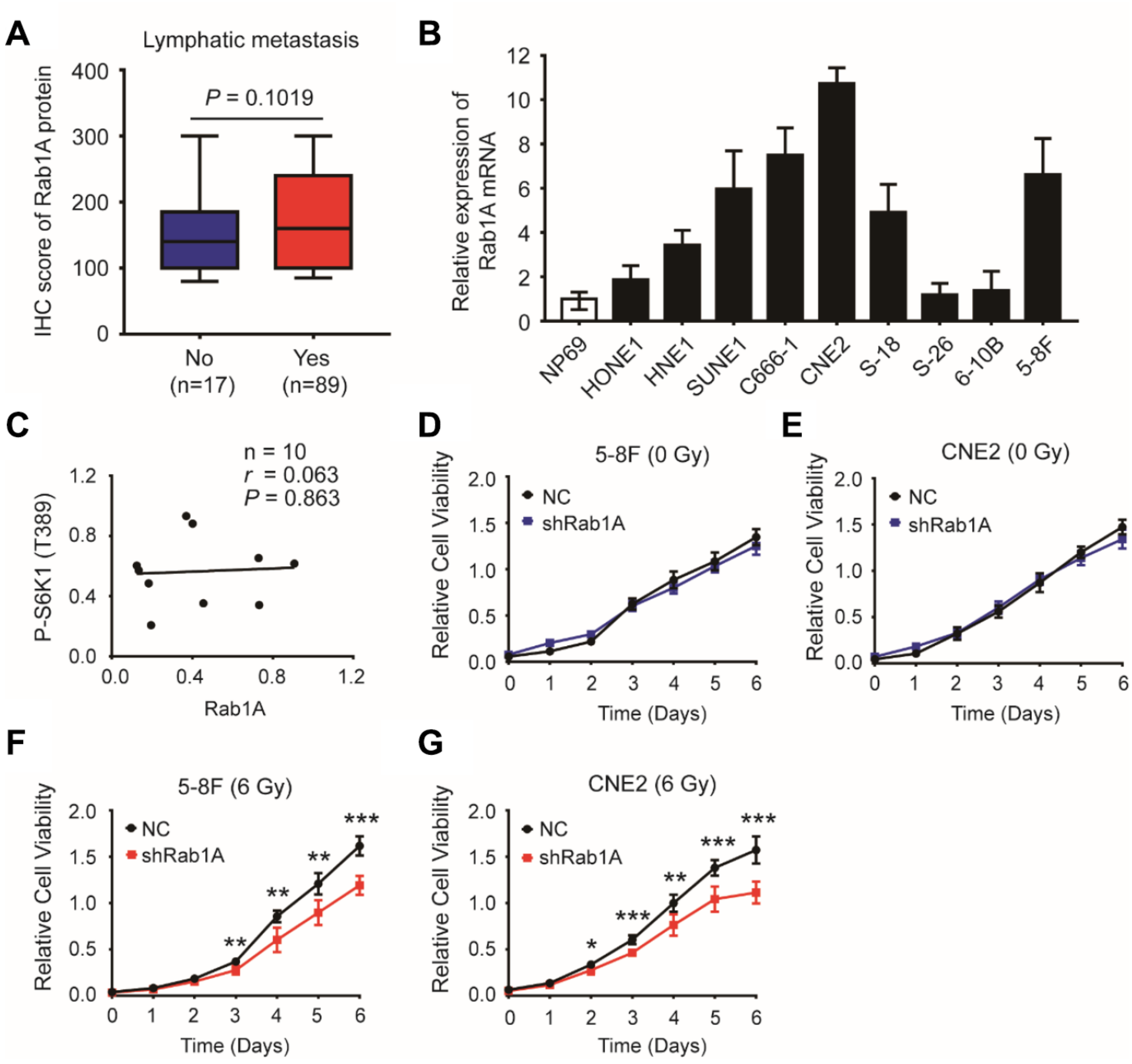

Supplementary Figure 1. (**A**) The expression of Rab1A protein in NPC patients with/without lymphatic metastasis. (**B**) Relative expression of Rab1A (normalized to GAPDH) in a panel of NPC cell lines and one human nasopharyngeal normal epithelium cell line NP69 was examined by RT-PCR. (**C**) The correlation between Rab1A and P-S6K1(T389) proteins in NPC cell lines was assessed by Spearman correlation assay. (**D** and **E**) CCK8 assay of 5-8F and CNE2 cells after knockdown of Rab1A before IR. (**F** and **G**) CCK8 assay were used to observe the effect of Rab1A knockdown on 5-8F and CNE2 cells proliferation after 6 Gy of irradiation as a single dose. Error bars: mean ± SD from three independent experiments. $^{*}P$ <0.05, $^{**}P$ < 0.01, $^{***}P$ < 0.001.

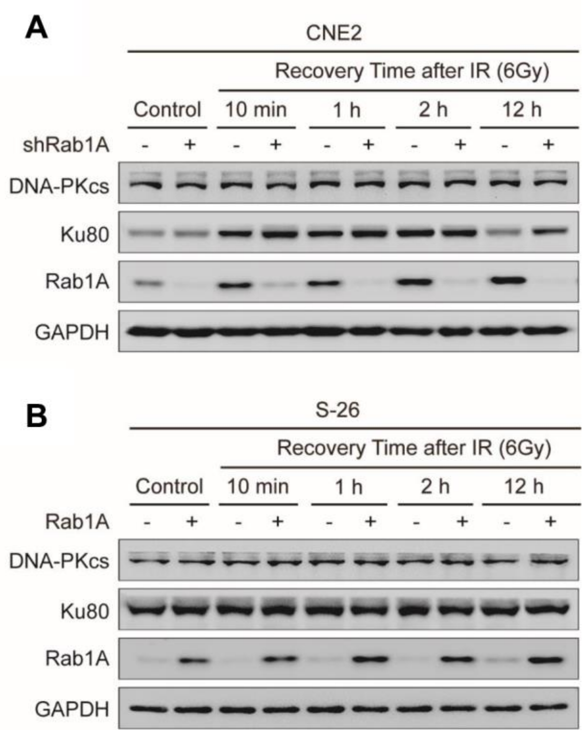

Supplementary Figure 2. (**A** and **B**) Western blotting analysis of representative proteins of NHEJ pathway-related proteins (Ku80 and DNA-PKcs) in the four groups of CNE2 and S-26 cells treated with or without 6 Gy of irradiation. GAPDH was used as a loading control. The results showed that aberrant expression of Rab1A did not affect the expressions of Ku80 and DNA-PKcs proteins in CNE2 or S-26 cells treated with/without radiation.
